# Molecular Mechanisms of Retinoid Receptors in Diabetes-Induced Cardiac Remodeling

**DOI:** 10.3390/jcm3020566

**Published:** 2014-06-04

**Authors:** Jing Pan, Rakeshwar S. Guleria, Sen Zhu, Kenneth M. Baker

**Affiliations:** Division of Molecular Cardiology, Department of Medicine, College of Medicine, Texas A & M Health Science Center, Baylor Scott & White Health, Central Texas Veterans Health Care System, Temple, TX, 76504, USA; E-Mails: rsguleria@medicine.tamhsc.edu (R.S.G.); szhu@medicine.tamhsc.edu (S.Z.); kbaker@medicine.tamhsc.edu (K.M.B.)

**Keywords:** diabetes mellitus, diabetic cardiomyopathy, retinoic acid, retinoic acid receptor, retinoid X receptor

## Abstract

Diabetic cardiomyopathy (DCM), a significant contributor to morbidity and mortality in diabetic patients, is characterized by ventricular dysfunction, in the absence of coronary atherosclerosis and hypertension. There is no specific therapeutic strategy to effectively treat patients with DCM, due to a lack of a mechanistic understanding of the disease process. Retinoic acid, the active metabolite of vitamin A, is involved in a wide range of biological processes, through binding and activation of nuclear receptors: retinoic acid receptors (RAR) and retinoid X receptors (RXR). RAR/RXR-mediated signaling has been implicated in the regulation of glucose and lipid metabolism. Recently, it has been reported that activation of RAR/RXR has an important role in preventing the development of diabetic cardiomyopathy, through improving cardiac insulin resistance, inhibition of intracellular oxidative stress, NF-κB-mediated inflammatory responses and the renin-angiotensin system. Moreover, downregulated RAR/RXR signaling has been demonstrated in diabetic myocardium, suggesting that impaired RAR/RXR signaling may be a trigger to accelerate diabetes-induced development of DCM. Understanding the molecular mechanisms of retinoid receptors in the regulation of cardiac metabolism and remodeling under diabetic conditions is important in providing the impetus for generating novel therapeutic approaches for the prevention and treatment of diabetes-induced cardiac complications and heart failure.

## 1. Introduction

Diabetic cardiomyopathy (DCM), characterized by alterations in cardiac morphology and function, independent of hypertension or coronary disease, is a significant contributor to morbidity and mortality associated with diabetes [1,2]. The etiology of DCM is multifactorial and incompletely understood. We and others have shown that chronically activated cardiac NF-κB, JNK pathways, impaired insulin signaling-induced metabolic disturbances, dysregulated calcium homeostasis and an over-activated renin-angiotensin system are emerging as major molecular and metabolic mechanisms for DCM [3,4,5,6,7,8]. There is no specific therapeutic strategy to effectively treat patients with DCM, due to a lack of mechanistic details.

Retinoic acids (RA), the active metabolite of retinol (vitamin A), are involved in the regulation of cell proliferation and differentiation, with effects being mediated by two classes of nuclear receptors, retinoic acid receptors (RAR) and retinoid X receptors (RXR). Retinoic acid signaling components (retinol binding protein 4, retinal, retinoic acid and retinoid receptors) have been implicated in modulating the development of obesity, insulin resistance and diabetes [9,10,11,12,13,14,15]. Whether retinoic acid and RAR/RXR-mediated signaling are of importance in the development of DCM is not well known. This review will focus on recent new developments in retinoic acid signaling and diabetes-induced cardiac remodeling.

## 2. Diabetic Cardiomyopathy

**Figure 1:**
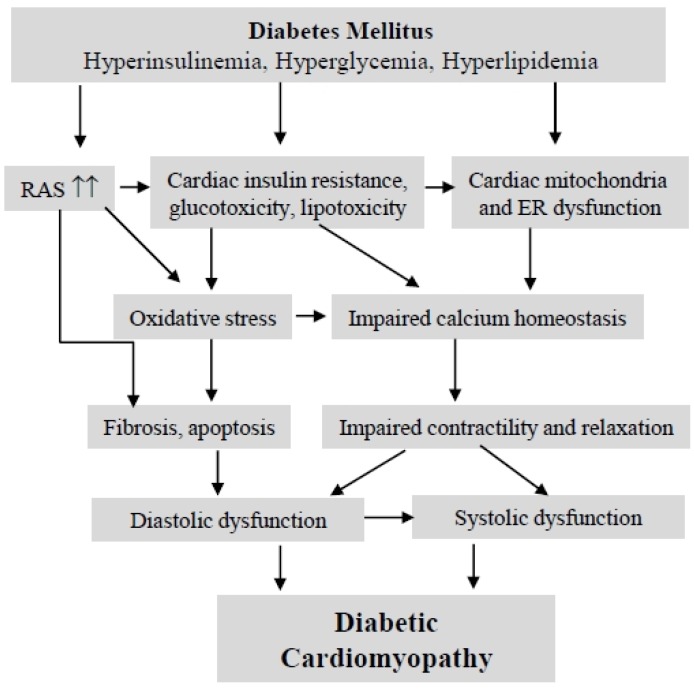
The pathophysiology of diabetic cardiomyopathy. Diabetes-induced cardiac insulin resistance, glucose/lipid dysmetabolism and activation of the renin-angiotensin system promote the development and progression of diabetic cardiomyopathy (DCM). ER, endoplasmic reticulum; RAS, renin-angiotensin system.

The epidemic of obesity and a sedentary lifestyle is projected to result in over 439 million individuals with diabetes mellitus (DM) worldwide by 2030 [16]. DM is an important and prevalent risk factor for congestive heart failure. Indeed, cardiovascular complications are the leading cause of diabetes-related morbidity and mortality (65%) [17]. Heart failure (HF) is a syndrome that represents the end stage of different forms of heart disease, affecting nearly 5.8 million people in the United States, and more than 23 million people worldwide [18]. HF results in high hospitalization rates and mortality; up to 40% of patients die within one year of their first hospitalization, resulting in significant health and financial burdens on patients and society [19,20]. The Framingham report indicates that diabetes increases the risk of heart failure, independent of other contributing risk factors [21,22]. The risk of heart failure in diabetic subjects was increased 2.4-fold in men and five-fold in women. This risk was independent of age, hypertension, obesity, coronary artery disease and hyperlipidemia. The impairment of left ventricular function in diabetic patients without underlying coronary artery disease or hypertension is now recognized as a distinct clinical entity termed “diabetic cardiomyopathy” [23], which was first described by Rubler *et al*., who reported data from four diabetic patients with heart failure without evidence of hypertension, coronary artery disease, valvular or congenital heart disease [24]. Increased left ventricle (LV) diastolic stiffness and relaxation disturbances are recognized as the earliest manifestation of diabetes-induced cardiac remodeling and have been noted in 27%–70% of asymptomatic diabetic patients [25,26]. Of note, left ventricular hypertrophy, which is a relevant prognostic risk factor in heart disease, occurred more frequently in diabetic patients and is a major contributor for the increased ventricular stiffness and diastolic dysfunction [27]. LV systolic dysfunction supervenes only at later stages of DCM. A decreased LV systolic ejection fraction (LVEF) provides a good reflection of the severity of systolic dysfunction and heart failure. Though the pathogenesis and pathophysiology of DCM is multifactorial and poorly understood, current evidence suggests that diabetes affects cardiac remodeling through a variety of mechanisms (Figure 1), including over-activation of the renin-angiotensin system, insulin resistance, metabolic disturbances (hyperglycemia, hyperinsulinemia and hyperlipidemia), fibrosis and impairment of calcium homeostasis [28,29,30]. Given the common and increasing prevalence of diabetes in the general population, diabetic cardiomyopathy is a significant and growing public health concern.

Diabetic patients may take several years to develop overt DCM. In the early stage, a majority of the cases of DCM are subclinical, and patients may not have overt symptoms or signs of disease. Thus, the implementation of diagnostic strategies for DCM to identify the disease at its early stages would be important in the prevention and early treatment of DCM. Echocardiography and Doppler imaging are able to detect significant cardiac abnormalities before the onset of symptomatic HF. Diastolic dysfunction, characterized as HF with normal EF, is present in 75% of asymptomatic diabetic patients [31]. The mitral inflow patterns, early ventricular filling wave (E-wave) and the late ventricular filling wave (A-wave), the E/A ratio, the isovolumetric relaxation time (IVRT), cardiac stiffness and myocardial tissue velocities during the cardiac cycle can be assessed by echocardiography and tissue Doppler imaging [32,33]. With more sensitive techniques, like strain deformation imaging, researchers observed that LV systolic function is impaired despite normal LVEF [34,35] and in 28% of patients with normal diastolic function, suggesting that systolic strain alteration may exist despite normal diastolic function and that diastolic dysfunction should not be considered the first marker of a preclinical form of DCM. Cardiac magnetic resonance imaging (MRI) and positron emission tomography (PET) have recently emerged as a highly sensitive imaging tool for detecting myocardial metabolic changes, LV wall motion abnormalities, geometry and cardiac hypertrophy [36] and may serve as a new technique in the diagnosis of DCM at an early stage.

Treatment should begin as soon as the diagnosis of diastolic dysfunction is made, to prevent further development of heart failure. The management and treatment of DCM have focused on changes in lifestyle, improving glycemic control, treatment of dyslipidemia and management of the coexistent hypertension, coronary artery disease (CAD) and heart failure [37]. Regular physical exercise and limitation of fat and total energy intake remain the cornerstone of the management of overweight diabetic patients. Clinical studies have shown that lifestyle changes with diet and/or exercise interventions led to a significant decrease in the incidence of DCM among subjects with type 2 diabetes [38,39]. Hyperglycemia can directly impair biological processes important for the maintenance of normal cellular function and serve as the prime driver behind diabetic DCM. Improvement of glycemic control has been shown to be associated with better outcomes in diabetic microvascular complications in clinical trials [40,41]. However, many prospective randomized clinical trials, such as ACCORD (Action to Control Cardiovascular Risk in Diabetes) and ADVANCE (Action in Diabetes and Vascular Disease: Preterax and Diamicron MR Controlled Evaluation), failed to conclusively demonstrate that aggressive glycemic control improves the cardiovascular prognosis of diabetic patients, especially those with advanced type 2 diabetes [41,42,43]. Different drug combinations, like metformin plus dipeptidyl peptidase-4 inhibitors/Glucagon-like peptide-1 mimetics, metformin plus pioglitazone, sulphonylurea or insulin, may be necessary for optimal glycemic control [37]. Treatment of dyslipidemia significantly reduces the incidence of major cardiovascular events, including DCM, in type 2 diabetic patients, and a reduction in low density lipoprotein cholesterol should be the primary goal for lipid modifying interventions [44,45]. Various vasoactive drugs, such as renin inhibitor, angiotensin convertase enzyme inhibitor (ACEI), angiotensin II receptor blocker (ARB), beta adreno-receptor blockers and calcium channel blockers, have been used in the management of hypertension, CAD and HF and have been found to be beneficial for patients with DCM [46,47]. ACEIs and ARBs have demonstrated cardiovascular protection in diabetic patients [48,49]. Aliskiren is the first representative of a new class of orally active direct renin inhibitors and provides a more complete blockade of the renin-angiotensin system (RAS), effectively suppressing residual angiotensin II production and the counter-regulatory increase in plasma renin activity observed in patients receiving monotherapy with ACEIs or ARBs [50]. However, the combination therapy of aliskiren with ACEIs or ARBs in diabetic hypertensive patients with chronic kidney disease is controversial [51,52]. Currently, a combination therapy of aliskiren with ACEIs and ARBs is not recommended in patients with type 2 diabetes mellitus and renal impairment. Beta-blockers have been shown to prevent and even reverse cardiac remodeling, resulting in improved LV function and a reduction in mortality in diabetic and non-diabetic patients [53]. Beta-blockers should be given to all diabetic patients with any evidence of HF, unless specifically contraindicated. Calcium channel blockers are capable of reversing the intracellular calcium defects and preventing diabetes-induced myocardial changes and are safe and effective as first-line or add-on therapies in diabetic hypertensive patients [54]. In addition to diabetic and heart failure therapies, other novel treatments are under investigation for DCM. Continued efforts to identify effective preventative strategies and treatments are essential.

## 3. Retinoic Acid and Retinoid Receptor Signaling

**Figure 2:**
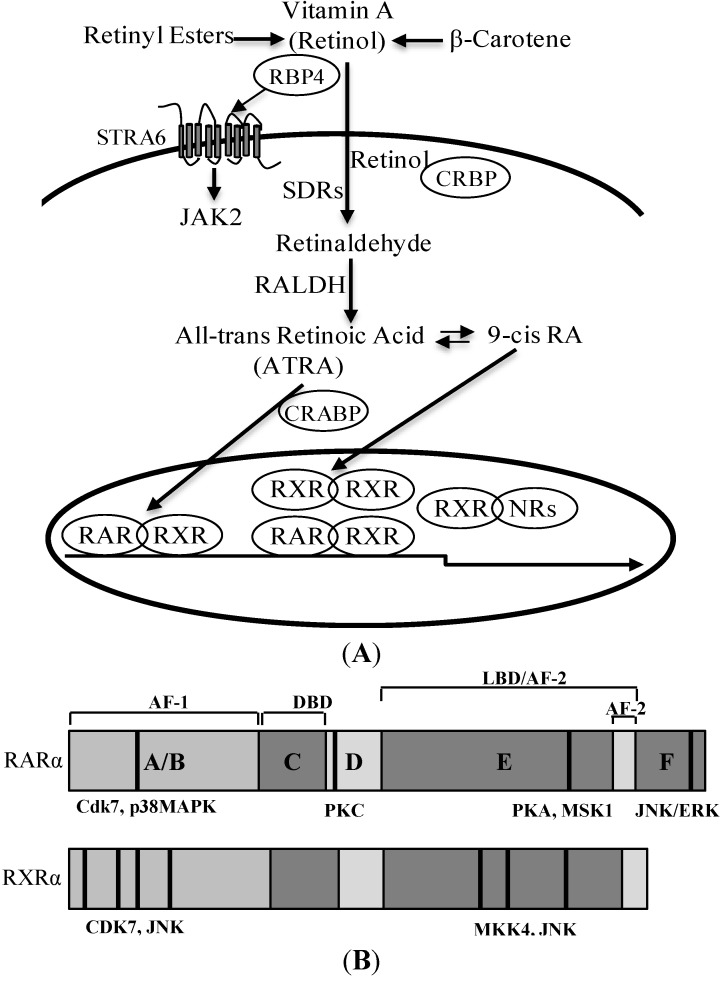
Retinoid metabolism and the structure of retinoid receptors. (**A**) Retinol binds to RBP4 (retinol binding protein 4) in plasma. RBP4 bonds to STRA6, resulting in the activation of the JAK2 signaling pathway. Retinol bound with cellular retinol-binding protein (CRBP) is converted to retinaldehyde by short-chain dehydrogenase/reductase (SDR); and then to all-*trans* retinoic acid (ATRA) by retinaldehyde dehydrogenase (RALDH); (**B**) Schematic representation of the functional domains and the major phosphorylation sites of RARα (retinoic acid receptor α) and RXRα (retinoid X receptors α). The DNA-binding domain (DBD) and the ligand-binding domain (LBD) are schematically represented (not to scale). The functional AF-1 and AF-2 domains lie in the A/B and E regions, respectively. Phosphorylation sites are shown in a bold black line.

Retinoic acid (RA), the active metabolite of vitamin A (Figure 2A), is an important signaling molecule in embryonic development and in regulating cell survival, differentiation and death [55]. The precursors of RA have to be obtained from the diet, either as retinyl esters from animal sources or β-carotene from plants. In the circulation, retinol binds to the specific transport protein, retinol-binding protein 4 (RBP4), and forms a complex with transthyretin to prevent renal filtration and catabolism [56]. Retinol is taken up by target cells from the circulation through spontaneous diffusion across plasma membranes, fluxes that are dictated by its extracellular to intracellular concentration gradient [57], or through a cell surface receptor, STRA6 (stimulated by retinoic acid) [58]. Recent studies have shown that STRA6 mediated retinol uptake is only required in eye, and it is not critical for maintaining retinol availability in tissues that express it [59,60]. Intracellularly, retinol associates with cellular retinol-binding protein (CRBP) and is oxidized to retinaldehyde (rate-limiting) by two types of enzymes: alcohol dehydrogenase (ADH) of the medium-chain ADH family and short-chain dehydrogenase/reductase (SDR) [61,62]. Studies have demonstrated that the ADH enzymes are not essential for RA biosynthesis from a physiologically relevant supply of vitamin A during embryogenesis or adulthood and that SDR family members have been implicated in the regulation of RA homeostasis [63,64]. Retinaldehyde is further oxidized to all-*trans* retinoic acid (ATRA), irreversibly by retinaldehyde dehydrogenase (RALDH) [65]. After binding to cellular retinoic acid binding proteins (CRABP), ATRA is transported into the nucleus and binds to nuclear receptors to regulate gene transcription or delivered to cytochrome (CYP) enzymes for degradation [66,67]. Most of the biological effects of RA are mediated by retinoic acid receptors (RAR) and retinoid X receptors (RXR). Both have three subtypes, α, β and γ, and are members of the nuclear hormone receptor super-family. RAR and RXR modulate gene expression by acting as ligand-dependent transcription factors. RARs primarily bind to ATRA, and RXRs bind to a stereoisomer, 9-*cis*-RA. Upon ligand binding, receptors form dimers and bind to DNA motifs known as RA response elements (RAREs), located in the regulatory regions of target genes and which subsequently modulate the transcription of an array of target genes [68,69] RAR is activated only when heterodimerizing with RXRs. RXR modulates gene transcription by forming either homodimers or heterodimers with several other nuclear receptors, including RAR, the vitamin D receptor, peroxisome proliferator activated receptors (PPARs), farnesoid X receptors, the liver X receptor (LXR) and the thyroid hormone receptor. Therefore, RXR plays a key role in nuclear signaling pathways involving its dimeric partners. RAR and RXR have conserved structures with six regions (A–F) (Figure 2B). The N-terminal A/B region contains another transcription activation domain (AF-1) that functions autonomously and ligand independently. This domain contains many phosphorylation sites and is the target of multiple kinases. The central C region is the most conserved DNA-binding domain (DBD). The C-terminal contains a ligand-binding domain (LBD) and ligand-dependent transactivation domain (AF-2). In the absence of a ligand, RXR/RAR heterodimers are bound to DNA in a complex with corepressors (such as NCoR (nuclear receptor co-repressor) and SMRT (silencing mediator of retinoid and thyroid hormone) receptors) that actively repress transcription. Upon ligand binding, co-repressors are released, and co-activator complexes (including p160 family members, the CREB-binding protein and p300) are recruited to activate transcription. The transactivation of RAR and RXR is also regulated by phosphorylation. Signaling pathways initiated by Cdk7 and PKA are involved in the positive control of retinoid-regulated transcription [70,71]. PKC, Akt and JNK-induced phosphorylation leads to degradation and transrepression of RAR/RXR [72,73,74]. Several lines of evidence indicate that phosphorylation of RAR/RXR has a crucial role in the development of certain cancers [75,76]. The requirement for retinoic acid during embryogenesis (brain, heart, lung and limb formation) has been long appreciated [77], and deregulated retinoid signaling contributes to serious diseases, such as cancer and metabolic diseases [78,79,80,81]. Retinoids have been investigated extensively for their use in cancer prevention and treatment [82].

## 4. Retinoic Acid Signaling Is Involved in the Development of Diabetes

**Figure 3:**
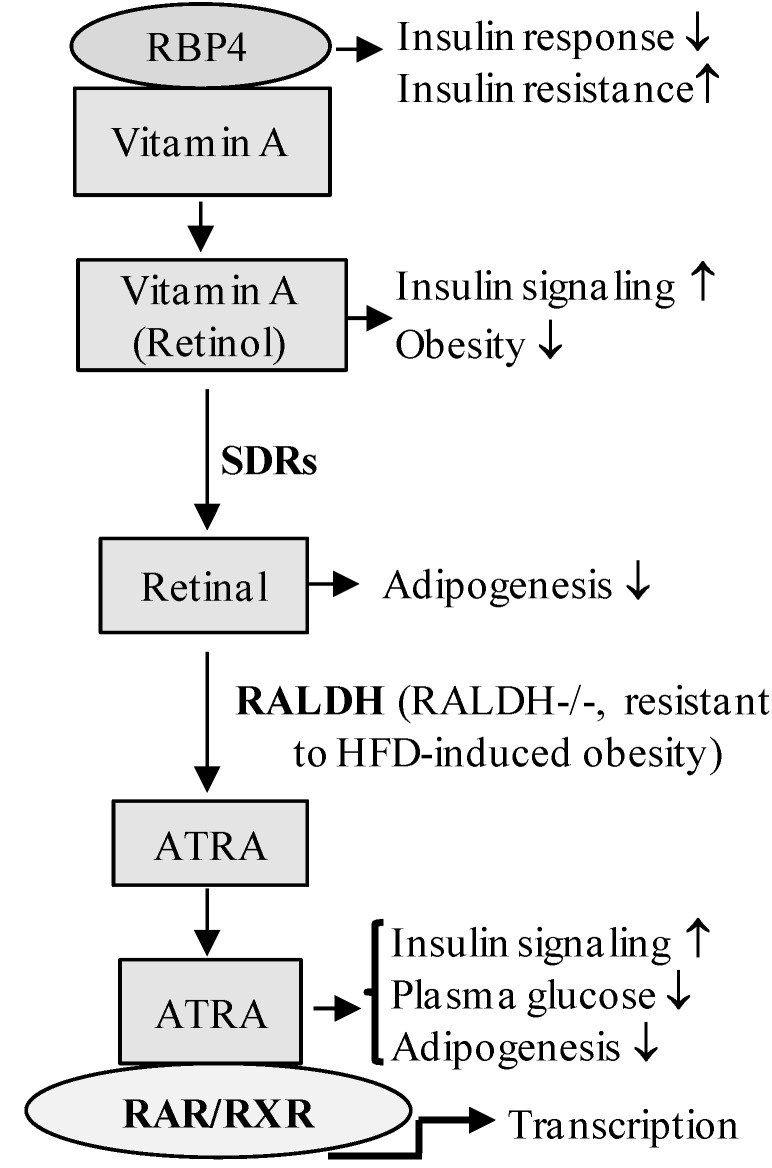
Retinoid-mediated signaling in the regulation of glucose/lipid metabolism. The schematic diagram represents the involvement of retinoid signaling components in the regulation of glucose/lipid metabolism in the development of obesity and diabetes. RBP4, retinol binding protein 4; SDR, short-chain dehydrogenase/reductase; RALDH, retinaldehyde dehydrogenase; HFD, high fat diet; RALDH−/−, RALDH knockout mice.

To date, the impacts of vitamin A and retinoids on the energy metabolism of the liver, adipocytes, pancreatic β-cells and skeletal muscle in animals and humans have been demonstrated in basic and clinical investigations (Figure 3) [14,15,83,84,85,86,87,88,89]. There is increasing evidence that vitamin A metabolism is impaired, especially in poorly controlled DM [90,91]. Studies have shown a decreased plasma level of retinol in type 1 diabetic patients and in streptozotocin-induced diabetic animal models [92,93] and that dietary supplementation of vitamin A inhibits the development of type 1 diabetes [94]. Van Y. H. *et al*. reported that ATRA inhibits the development of type 1 diabetes by T regulatory-dependent suppression of interferon γ-producing T-cells [95], suggesting that the use of ATRA to induce immune tolerance may provide an effective method to inhibit type 1 DM. Although the plasma level of retinol is normal in type 2 DM, an increased serum level of RBP4 is observed. The increased serum RBP4 concentrations in type 2 diabetic patients may be associated with diabetes-related renal dysfunction, cardiovascular disease and imbalances in lipid metabolism [83,96,97]. Lowering the RBP4 level by ATRA or a synthetic retinoid, fenretinide, leads to improved insulin action and glucose tolerance in insulin-resistant obese mice [91,92,98,99,100]. Recent studies have shown that holo-RBP/STRA6 is involved in the regulation of activation of the JAK2/STAT5 cascade and insulin resistance, by inducing the suppressor of cytokine signaling 3 (SOCS3) and PPARγ expression [59,101]. RBP4-mediated inhibition of insulin signaling is also regulated by promoting the expression of proinflammatory cytokines through a toll-like receptor 4- and c-Jun N-terminal kinase (JNK)-dependent and retinol-independent mechanism [10]. Activation of RAR/RXR-mediated signaling also has anti-diabetic effects in type 2 DM. In 1997, Mukherjee R. *et al*. first reported that a synthetic activator of RXR functions as an insulin sensitizer, markedly decreasing serum glucose and insulin levels in mouse models of type 2 diabetes and obesity [102]. Treatment of diabetic (db/db) mice with LG268 (selective RXR ligand) significantly increased insulin-stimulated glucose transport in skeletal muscle, via increasing insulin-stimulated insulin receptor substrate 1 (IRS1) tyrosine phosphorylation and AKT phosphorylation [103]. Our group also reported that ATRA, Am580 (RARα selective ligand) and LGD1069 (RXR ligand) lowered the blood glucose level and improved insulin resistance in a Zucker diabetic fatty (ZDF) rat type 2 diabetic model [6,7].

Obesity has historically been shown to be a risk factor for type 2 diabetes. During the past 50 years, both clinical and animal studies have suggested the involvement of retinoids and RAR/RXR signaling in the regulation of lipid metabolism and obesity. Supplementation of obese rats with high levels of dietary vitamin A reduced body weight gain and visceral adiposity and improved insulin sensitivity, by decreasing soleus muscle PTP1B (protein tyrosine phosphatase 1B) levels and increasing insulin receptor phosphorylation [104]. Retinoic acid treatment also resulted in weight loss, decreased adiposity and increased insulin sensitivity, through promoting lipid oxidation capacity in skeletal muscle and liver in several lean and/or obese mouse models [89,105,106]. Similarly, a diet deficient in vitamin A led to an increase in body weight and adiposity in mice [107,108]. Studies have shown that ATRA represses obesity and insulin resistance in a type 2 diabetic animal model, through activation of proliferation-activated receptor δ (PPARδ) [13]. ATRA-induced body fat loss correlates with the activation of brown adipose tissue, reduced adipogenic/lipogenic capabilities, increased capabilities for oxidative metabolism in white adipose tissue depots and increased lipid oxidation capacity in skeletal muscle and adipocytes [106,109]. Retinoic acid synthesizing enzymes (RALDH) are also involved in the regulation of lipid metabolism and high fat diet-induced insulin resistance [110]. The polymorphism in the promoter of the *CRABP2* gene is associated with increased plasma low-density lipoprotein cholesterol (LDL-C) concentrations in familial hypercholesterolemia patients [111]. These studies indicate that altered expression/activation of RA signaling closely correlates with the development of DM and insulin resistance.

## 5. Retinoid Receptor-Mediated Signaling and Diabetic Cardiomyopathy

### 5.1. Retinoid Receptor-Mediated Signaling in Cardiac Remodeling

Cardiac remodeling has a major role in the progression to HF, which is associated with alterations in the ventricular structure and function, resulting from myocardial injury, pressure or volume overload. At the cellular level, cardiac remodeling is characterized by cardiomyocyte hypertrophy, fibroblast hyperplasia accompanied by an increase in collagen deposition within the interstitial matrix (fibrosis) and cell death. It is well known that RA signaling is required for cardiac development [112,113,114,115]. Changes in RA homeostasis (lacking or excess of RA) result in severe malformations during cardiogenesis, suggesting that a precise tissue concentration of RA is indispensable for the proper induction of signaling pathways important for normal myocardial cell growth and differentiation in early embryonic stages. RA signaling has been implicated in the regulation of cell differentiation, proliferation and apoptosis [116,117], and a substantial body of knowledge has accumulated on its role in the regulation of cardiomyocyte growth, apoptosis and cellular function in response to various pathophysiological stimuli. Using an *in vitro* cultured cardiomyocyte model, we and others have demonstrated that RA suppresses myocardial cell growth in response to hypertrophic stimuli, including cyclic stretch, angiotensin II (Ang II), endothelin-1 and phenylephrine [118,119,120,121]. Ang II-induced cardiac fibroblast growth and collagen secretion are also inhibited by RA [122]. Chronic RA treatment attenuated tobacco smoke exposure-induced cardiac hypertrophy and LV dysfunction in rats [123] and prevented medial thickening of intramyocardial and intrarenal arteries and ventricular fibrosis during the development of hypertension in SHR (Spontaneously Hypertensive) rats [124]. In a rat model of myocardial infarction, coronary occlusion-induced LV morphological (hypertrophy) and functional changes were improved by RA treatment [125]. Using a chronic rat pressure-overload model, our group demonstrated that RA inhibits cardiomyocyte apoptosis and fibrosis and improves both systolic and diastolic heart function, through the inhibition of the oxidative stress-induced activation of MAP kinase pathways and the expression of renin-angiotensin system (RAS) components [126]. These studies suggest that activation of the RAR/RXR signaling pathway has an important role in preventing the transition from compensatory hypertrophy to heart failure.

Diabetic cardiomyopathy represents a distinct structural and functional disorder of the myocardium characterized by cardiac hypertrophy and diastolic heart failure at the early stage, progressing to overt systolic heart failure at the later stage. Using *in vitro* primary cultured cardiomyocytes, we have demonstrated that high glucose-induced apoptosis and reactive oxygen species (ROS) generation was prevented by both RAR and RXR agonists. Silencing the expression of RARα and RXRα, by small interference RNA, promoted apoptosis under normal conditions and significantly enhanced high glucose-induced apoptosis [8]. Using an *in vivo* type 2 diabetic animal model, we provided additional evidence supporting a role of RARα and RXRα in the diabetes-induced development of cardiac remodeling. We have shown that chronic treatment with the RARα selective ligand, Am580, and the RXR selective ligand, LGD1069, significantly improved diastolic dysfunction and attenuated LV hypertrophy, fibrosis, apoptosis and inflammatory responses in ZDF rats [7]. Our data indicate that RA/RAR/RXR-mediated signaling may serve as an alternative therapeutic target in the prevention and treatment of diabetic cardiomyopathy.

### 5.2. Mechanisms Involved in Retinoid Receptor Signaling Regulated Diabetic Cardiomyopathy

#### 5.2.1. Activation of RAR/RXR Signaling Improves Cardiac Insulin Signaling and Glucose/Lipid Metabolism

In type 1 and type 2 DM, glucose uptake, glycolysis and pyruvate oxidation are impaired. Additionally, a lack of insulin function augments lipolysis and the release of fatty acids (FA) from adipose tissue. Derangements in cardiac lipid and glucose metabolism are becoming recognized as an early event in the deterioration of heart function in diabetes. Hyperglycemia is the main driving force at all stages of diabetic cardiomyopathy. It triggers various adaptive and maladaptive responses, which lead synergistically to the development of heart failure [29,127]. Under normal physiological conditions, glucose is one of the major carbohydrates utilized by the heart. In type 1 diabetic animals, reduced *GLUT* gene and protein expression compromises cardiac glucose uptake and oxidation [128]. In obese or type 2 diabetic animals, cardiac glucose uptake is reduced as a consequence of reduced GLUT4 protein and impaired insulin signaling [129,130]. The impaired glucose uptake forces cardiomyocytes to rely less on glucose metabolism and more on β-oxidation of free fatty acids (FA) for energy production. This dramatic shift results in the loss of metabolic flexibility and decreased cardiac efficiency in diabetic heart. Moreover, augmented FA oxidation increases lipid deposition and promotes insulin resistance. Lipid accumulation within cardiomyocytes is associated with impaired contractile function [131]. FA metabolism also generates a number of toxic intermediates that accumulate in myocardial cells, resulting in lipotoxicity, and contributes to the initiation and development of diabetic cardiomyopathy [132,133,134,135,136]. Oxidative stress, the imbalance between reactive oxygen species production and the breakdown by endogenous antioxidants, has been implicated in the onset and progression of cardiovascular diseases, such as congestive heart failure and diabetic cardiomyopathy [137,138]. Studies have shown that hyperglycemia significantly enhances free radical formation and mitochondrial generation of superoxide, which increases oxidative stress and activates cellular signal transduction and cardiac cell death, leading to diabetic cardiomyopathy [8,22,139,140]. Increased lipid accumulation and FA β-oxidation in diabetic myocardium also leads to overwhelming generation of ROS, which is associated with impaired insulin signaling and the development of heart dysfunction [141,142]. As such, insulin resistance, increased FA and hyperglycemia can be considered triggers for the cardiac phenotype in diabetes. An understanding of the cellular effects and mechanisms of these metabolic disturbances on cardiomyocytes will be important in predicting the structural and functional cardiac consequences and in developing therapeutic approaches for the treatment of DM-induced cardiac remodeling.

A number of studies have shown that RA lowers plasma glucose levels and improves insulin resistance in adipocytes, skeletal muscle and liver tissues [14,15,83,84,85,86,87,88,89]. Our group further provided evidence that the activation of RAR/RXR signaling improves cardiac glucose metabolism in ZDF rats [7]. We demonstrated that activation of RARα and RXR by Am580 and LGD1069 not only improved systemic glucose homeostasis, but also had a significant effect on cardiac glucose metabolism. Impaired Akt/GSK3β insulin signaling and decreased gene expression of GLUT1, GLUT4, aldolase A and hexokinase 2, in ZDF rat hearts, was improved by Am580 or LGD1069 treatment, indicating that the activation of RAR and RXR signaling rescued the impaired cardiac insulin signaling and promoted glucose transportation and utilization. Diabetes-induced cardiac oxidative stress, apoptosis and activation of MAP kinases and NF-κB pathways were inhibited by Am580 and LGD1069. Thus, it is likely that the beneficial effect of Am580 or LGD1069 on diabetic cardiomyopathy is mediated at least partially by reducing glucotoxicity-induced cardiac oxidative stress and activation of apoptotic signaling. RAR and RXR-mediated signaling has an important role in the regulation of lipid metabolism and the development of obesity. However, the functional role of RAR and RXR in the regulation of cardiac lipid metabolism remains unclear. Previous studies have shown that ATRA suppresses hyperlipidemia and obesity and blocks adipogenesis, by enhancing FA oxidation and energy dissipation, through ATRA-induced activation of PPARβ/δ and RAR in adipocytes and liver [13,106]. We observed that ATRA suppressed body weight gain and increased cardiac FA β-oxidation in ZDF rats, following two weeks of treatment. Am580 had a similar effect on inhibiting body weight gain, cardiac FA uptake, β-oxidation and intracardiac lipid accumulation in ZDF rats, following 16 weeks of treatment [7]. These data suggest that ATRA and Am580 may alter substrate metabolism in diabetic heart, through rebalancing the utilization between glucose and FA, which further leads to improvement in cardiac efficiency and function. Though Am580 had a favorable effect on cardiac lipid metabolism, it had no effect on the increased plasma cholesterol and triglyceride levels. Am580, a selective agonist of RARα, is not an activator of PPARβ/δ, and thus, the mechanisms whereby Am580 regulates cardiac lipid metabolism and suppresses obesity may be different than those of ATRA. Compared to ATRA and Am580, LGD1069 further promoted body weight gain and hyperlipidemia following 16 weeks of treatment and significantly increased intracardiac lipid deposition in ZDF rat hearts. This is not consistent with previous studies, which have shown that RXR ligands, including LGD1069, decrease triglycerides and increase HDL levels in db/db or ob/ob mice [102,143]. It has been described that RXR can form permissive heterodimers with PPARs, the farnesoid-X-receptor and liver-X-receptors (LXR) and that these can be activated by both RXR-specific and partner-specific ligands [144]. We have observed that PPARα and LXR are activated by LGD1069 and that these receptors are also involved in the regulation of lipid metabolism [145,146]. The effect of LG1069 on cardiac lipid homeostasis we observed may be regulated not only through activation of RXR, but also PPAR and LXR. Therefore, studies with a receptor-subtype-specific ligand and receptor knockout models are critical to understand the downstream mechanisms involved in RAR/RXR-mediated metabolic signaling pathways.

#### 5.2.2. RAR/RXR Signaling in the Regulation of Cardiac NF-κB-Mediated Inflammatory Responses

Cardiac inflammation is one of the diverse mechanisms that are involved in the progression of diabetic cardiomyopathy [147,148,149]. Sustained increases in the levels of pro-inflammatory cytokines and chemokines, such as tumor necrosis factor-α (TNF-α), monocyte chemoattractant protein-1 (MCP-1) and interleukin-6 (IL-6), could directly and indirectly cause cardiac tissue injury, such as myocardial fibrosis, necrosis and apoptosis, which inevitably leads to cardiac diastolic and subsequent systolic dysfunction [150,151]. Pro-inflammatory cytokine expression is under the control of the ubiquitous and inducible transcription factor, NF-κB (nuclear factor-kappa B), which is activated by a variety of pathological stimuli, such as inflammatory cytokines, hyperglycemia, elevated free fatty acid levels in plasma, oxidative stress, angiotensin II, lipoproteins and anoxia [152,153]. Inactive NF-κB is primarily located in the cytoplasm in association with the inhibitor of κB (IκB). Following exposure to various stimuli, IκB is phosphorylated by IκB kinases (IKKs) and degraded by the ubiquitin proteasome pathway. A large body of evidence suggests that NF-κB activation occurs in a sustained manner in diabetes, in association with elevated blood glucose levels and inflammation [154,155,156,157]. The involvement of NF-κB in diabetic cardiomyopathy has been demonstrated in several studies on diabetic rat hearts [6,7,158,159]. Retinoids act as potent anti-inflammation agents that exert beneficial responses in the cardiovascular system [160,161]. Vitamin A and its metabolites inhibit several types of inflammatory reactions [162,163], and activated NF-κB signaling is associated with an increased inflammatory response in vitamin A deficiency [164,165,166]. The activation of RXR inhibits high glucose-induced upregulation of inflammation, by suppressing the activation of the NADPH oxidase-NF-κB pathway in human endothelial cells [167]. These data suggest that there is an interaction between RAR/RXR signaling and the NF-κB-mediated inflammatory pathway. We have demonstrated that the activation of RAR and RXR inhibits the activation of NF-κB and NF-κB-mediated gene expression of IL-6, TNF-α and MCP-1, *in vitro* and *in vivo* [6,7], indicating that the protective effects of RAR and RXR ligands on cardiomyocytes are mediated (at least in part) through the regulation of NF-κB signaling. The molecular mechanisms by which RA inhibits the activation of NF-κB appears to involve the inhibition of phosphorylation of IKK/IκBα and degradation of IκBα, through activation of PP2A (protein phosphatase 2A). The interactions between RAR/RXR and NF-κB signaling may have important implications in understanding the mechanisms involved in the development of diabetic cardiomyopathy.

#### 5.2.3. RAR/RXR Signaling in the Regulation of the Renin-Angiotensin System

The renin-angiotensin system (RAS) and its primary effector peptide, angiotensin II (Ang II), is a key regulatory system in blood pressure and volume homeostasis and has an essential role in the pathophysiology of heart failure. The RAS has been an important drug target for therapeutic intervention: Ang II receptor blockers (ARBs), angiotensin converting enzyme inhibitors (ACEi) and renin inhibitors reduce heart failure-related morbidity and mortality [168,169]. Accumulating evidence suggests that RAS blockade also has favorable effects on the parameters of glucose metabolism and the incidence of diabetes [170]. Expression/activation of the RAS has been implicated in the development of diabetic cardiomyopathy. Abnormal production of Ang II is associated with a high index of cardiomyocyte apoptosis in human and animal models of diabetes [171,172]. Hyperglycemia stimulates cardiomyocyte production of Ang II via an upregulation of most of the cellular components of the RAS [172,173,174,175,176]. The interaction between RA signaling and the RAS has previously been reported [126,177,178,179]. RA negatively regulates renal RAS components in rats with experimental nephritis [177]. Downregulation of AT_1_R mRNA and the repressed Ang II-stimulated AT_1_R promoter activity are observed in RA-treated vascular smooth muscle cells [178,180]. It has been shown that RA downregulates the expression of AT_1_R and upregulates ACE2 in SHR hearts, which is accompanied by a decrease in blood pressure [179,181]. Our group also demonstrated that the increased plasma level of Ang II, upregulated gene expression of cardiac and renal renin, angiotensinogen (AGT), ACE and AT_1_R are inhibited by ATRA in pressure overloaded rats and mechanically stretched cardiomyocytes [126], indicating that RA signaling is involved in regulating RAS components during the development of cardiac remodeling. We have reported recently that hyperglycemia-induced apoptosis and ROS production are inhibited by the AT_1_R blocker, indicating that Ang II-activated AT_1_R signaling contributes to hyperglycemia-induced cellular injury [8]. ATRA inhibited high glucose- and Ang II-induced cell apoptosis and ROS production. High glucose-induced gene expression of AGT, renin, AT_1_R and intracardiac Ang II production were significantly inhibited by ATRA. On the other hand, silencing the expression of RARα or RXRα increased the basal level of the gene expression of AGT and dramatically increased AGT expression in combination with high glucose stimulation. Our results suggest that a certain level of expression of RARα and RXRα is required in the regulation of the expression of AGT, under normal and hyperglycemic conditions, and that RARα/RXRα heterodimer-mediated signaling negatively regulates RAS components and AT_1_R-mediated signaling events. By inhibiting the rate-limiting step in the renin-angiotensin cascade, RA signaling might have some advantages over the RAS inhibitors widely used in the clinic.

#### 5.2.4. Does Impaired RAR/RXR Signaling Contribute to the Development of DCM?

The high degree of conservation of RAR and RXR across vertebrates and specific patterns of expression during embryogenesis and in adult tissues suggests that each receptor performs a specific function [182]. Targeted disruption of RAR and RXR, mainly RARα and RXRα in the embryonic stage, resulted in early postnatal or embryonic lethality and heart failure [183,184], suggesting that RARα and RXRα are the two main receptor subtypes that are involved in the regulation of cardiomyocyte differentiation and function. It has been reported that decreased cardiac expression of RXRα is involved in the altered myocardial metabolic phenotype in severe heart failure and that downregulation of RXRα may be responsible for impairment in free fatty acid oxidative pathways in the failing heart [185,186]. We have recently demonstrated that nuclear expression of RARα and RXRα was significantly downregulated in high glucose-stimulated cardiomyocytes and in diabetic rat myocardium [7,8,187]. High glucose stimulation also repressed physiological doses of RA-induced transcriptional activity of RAR and RXR, suggesting that a relative “RA-resistance” developed in response to hyperglycemia. Whether the alteration of RAR/RXR signaling is directly associated with the development of diabetes-induced cardiac remodeling remains unknown. We have observed that silencing the expression of RARα and RXRα in cardiomyocytes promotes high glucose-induced expression/activation of cardiac RAS components and apoptosis *in vitro* [8]. To further understand the role of RARα and RXRα in the regulation of cellular function in adult cardiomyocytes, in response to pathological stimuli, we generated tamoxifen-inducible RARα and RXRα cardiac-specific knockout mice (RARαKO and RXRαKO). Cardiac-specific gene deletion of RARα and RXRα results in diastolic dysfunction, which is associated with increased oxidative stress and apoptosis [188]. These results suggest that RARα and RXRα-mediated signaling is required to preserve normal cardiomyocyte function and that impairment of RAR/RXR signaling may accelerate the development of heart failure in response to pathological stimuli. Understanding the mechanisms underlying the impaired cardiac expression/activation of RARα and RXRα will be important in determining the novel mechanisms leading to heart failure.

**Figure 4:**
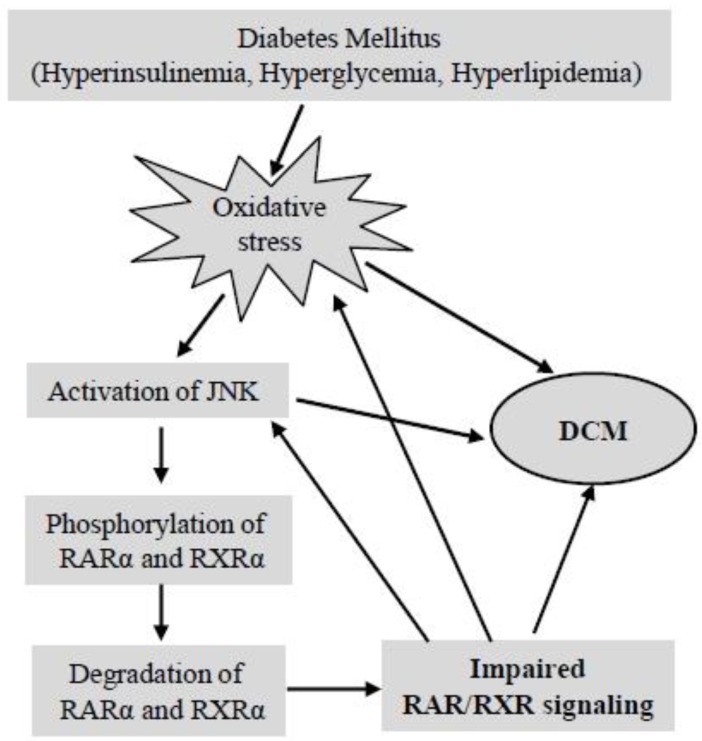
Schematic of the interaction between diabetes mellitus (DM), JNK and impaired RAR/RXR signaling in DCM. Mechanisms of DM-induced oxidative stress and JNK activation in the regulation of phosphorylation/transcriptional inhibition of RARα and RXRα and the development of DCM are proposed.

Retinoid receptor transcriptional activity is regulated by factors both intrinsic and extrinsic to the receptor complex. Although ligand binding is thought to be the primary means of activation of RAR and RXR, the transcriptional activity of RAR and RXR is also modulated by protein kinase-mediated phosphorylation and degradation [189]. RARα and RXRα contain multiple phosphorylation sites (Figure 2) and are substrates for a variety of serine/threonine kinases, including PKA, PKC, Cdk7 and MAP kinases [70,72,190,191,192]. Phosphorylation of RXRα at serine 260, a consensus MAP kinase site, results in the attenuation of ligand-dependent transactivation by the vitamin D_3_ receptor/RXRα complex [193]. Stress-induced phosphorylation of RXRα, through MAPK kinase 4 (MKK4) and JNK, results in the suppression of retinoid signaling in COS-7 cells (derived from CV-1 simian cells transformed by an origin-defective mutant of SV40) [74,194]. JNK activation by oxidative stress suppresses retinoid signaling through proteasomal degradation of RARα in hepatic cells [195]. These data suggest that oxidative stress/MAP kinases-regulated phosphorylation/degradation of RAR and RXR may have an important role in pathological stimuli-induced impairment of RAR/RXR signaling. We have recently reported that high glucose induces serine phosphorylation of RARα and RXRα in cardiomyocytes and that inhibition of intracellular ROS generation and activation of the JNK pathway prevents the downregulated expression and transcriptional repression of RARα and RXRα in cardiomyocytes. On the other hand, H_2_O_2_ stimulation or activation of JNK suppressed the expression and ligand-induced promoter activity of RARα and RXRα [187]. Based on these data, it is likely that diabetes-induced oxidative stress and activation of JNK promotes degradation and transcriptional inhibition of RARα and RXRα, through phosphorylation of RARα and RXRα at specific phosphorylation sites. Interestingly, silencing the expression of RARα and RXRα in cardiomyocytes promoted the activation of the JNK pathway *in vitro* [187] and *in vivo* (in the hearts of RARαKO and RXRαKO mice) [188], suggesting that impaired RAR/RXR signaling and oxidative stress/the JNK pathway form a vicious circle, which may significantly contribute to diabetes-induced cardiac remodeling (Figure 4). Identifying the phosphorylation site that is specifically linked to JNK-mediated degradation and transcriptional inhibition of RARα and RXRα will have functional significance in understanding the mechanism of DCM and in developing a therapeutic strategy for management.

## 6. Conclusions

Retinoic acid and RAR/RXR-mediated signaling are increasingly recognized as mediators of diabetes and obesity. Impaired RAR/RXR signaling may contribute to the development of diabetic cardiomyopathy and diastolic heart failure. Experimental data have provided strong evidence that RAR and RXR function as important transcriptional regulators in the development of diabetes-induced cardiac remodeling and heart failure, through the regulation of the cardiac renin-angiotensin system, glucose/lipid metabolism and oxidative stress associated signaling pathways. Therefore, retinoic acid and RAR/RXR-mediated signaling may represent a novel target for developing therapeutic approaches for the treatment and prevention of diastolic heart failure and DCM.
